# ﻿*Thismiamalayana* (Thismiaceae), a new mycoheterotrophic species from Peninsular Malaysia

**DOI:** 10.3897/phytokeys.242.120967

**Published:** 2024-05-31

**Authors:** Mat Yunoh Siti-Munirah, Chin Hardy-Adrian, Sharipudin Mohamad-Shafiq, Zainuddin Irwan-Syah, Abd Halim Hamidi

**Affiliations:** 1 Forest Research Institute Malaysia, 52109 Kepong, Selangor, Malaysia; 2 698 Persiaran Merak, Taman Paroi Jaya, 70400 Seremban, Negeri Sembilan, Malaysia; 3 Kampung Perlok, Ulu Cheka, 27030 Jerantut, Pahang, Malaysia; 4 Kampung Som, Damak, 27030 Jerantut, Pahang, Malaysia; 5 Jabatan Perhutanan Negeri Sembilan, 70503 Seremban, Negeri Sembilan, Malaysia

**Keywords:** Negeri Sembilan, new species, Pahang, *Thismia* subsect. *Odoardoa*, vulnerable

## Abstract

A new species of the mycoheterotrophic genus *Thismia* from Malaysia is described and illustrated. *Thismiamalayana* introduced here was found in two localities: in the lowlands of Gunung Angsi Forest Reserve, Negeri Sembilan, and in the hilly dipterocarp forests of Gunung Benom in Tengku Hassanal Wildlife Reserve, Pahang. *Thismiamalayana* falls into the section Thismiasubsect.Odoardoa, as it has creeping vermiform roots and free equal tepals. It is characterised by the following taxonomically important features: a sepia-brown, urceolate-curved floral tube, free equal tepals with terminal appendages, prominent bright yellow annulus and bright violet-blue stamens each bearing five appendages (one pair of club-shaped inwards-pointing, one pair of acute outwards-pointing, and one central appendage). According to the categories and criteria of the IUCN Red List, *T.malayana* is provisionally classified as Vulnerable.

## ﻿Introduction

The tropical rainforests of Southeast Asia together form the second largest area of tropical rainforests in the world. Also, the tropical lowland evergreen rainforest is the most species-rich forest formation in the region and structurally the most complex one, with species composition varying according to local soil and drainage conditions (Davis et al. 1995). Native plants of this type of forest include *Thismia* Griff., a genus of non-photosynthetic, mycoheterotrophic plants belonging to the family Thismiaceae. The genus *Thismia* comprises around 100 currently recognised species, and its range extends across various regions of the world, including tropical and subtropical Asia, northern and eastern Australia to New Zealand, the north-central USA, Costa Rica and southern tropical America (POWO 2024).

One of the most remarkable characteristics of *Thismia* is its mycoheterotrophic way of life. Unlike most plants, its species do not produce chlorophyll and are not capable of photosynthesis. A mycoheterotrophic plant is in fact a parasite of the mycorrhizal symbiosis, cheating it of the carbon resources that are shared in the mycorrhizal symbiosis, and this adaptation allows it to survive in the undergrowth of forests where the light availability is low (Merckx 2013; Watkinson 2016). *Thismia* species are known for their small size, which is usually only a few centimeters (Leake 1994). They are typically hidden in leaf litter and grow near tree roots or old rotten logs. Despite their small size, they are very sensitive to environmental changes, e.g. they decay easily when pulled out of its original habitat. *Thismia* species exhibit a number of unusual morphological features that continue to fascinate due to their amazing diversity of flower shapes and colours (Feller et al. 2022). The flower structure of *Thismia* is highly specialised to pollination by fungus gnats or other small insects (Thorogood and Siti-Munirah 2021). The flowers often have a complex shape with cup-like or spherical structures and intricate patterns that presumably attract pollinators. Due to their cryptic appearance, many *Thismia* species are still poorly known in terms of taxonomy and other biological aspects.

Another *Thismia* species is described here that is new to science. It was first discovered in 2020 in the Tengku Hassanal Wildlife Reserve (WR) (formerly known as Krau Wildlife Reserve) in the state of Pahang in Malaysia. It was later found also in a forested area in Ulu Bendul Recreation Park (RP), a popular nature and recreation area in Negeri Sembilan, Malaysia. It is located near the town of Kuala Pilah, about 25 kilometres from Seremban. The Ulu Bendul RP is also part of the Gunung Angsi Forest Reserve (FR) and offers nature lovers, hikers and explorers the opportunity to immerse themselves in the beauty of the tropical rainforest and discover its rich flora and fauna as well as its contribution to wildlife conservation. The type material of the new species was collected in February 2023 at the edge of the Gunung Angsi main trail in the forest area of Ulu Bendul RP.

## ﻿Materials and methods

**Map 1.**
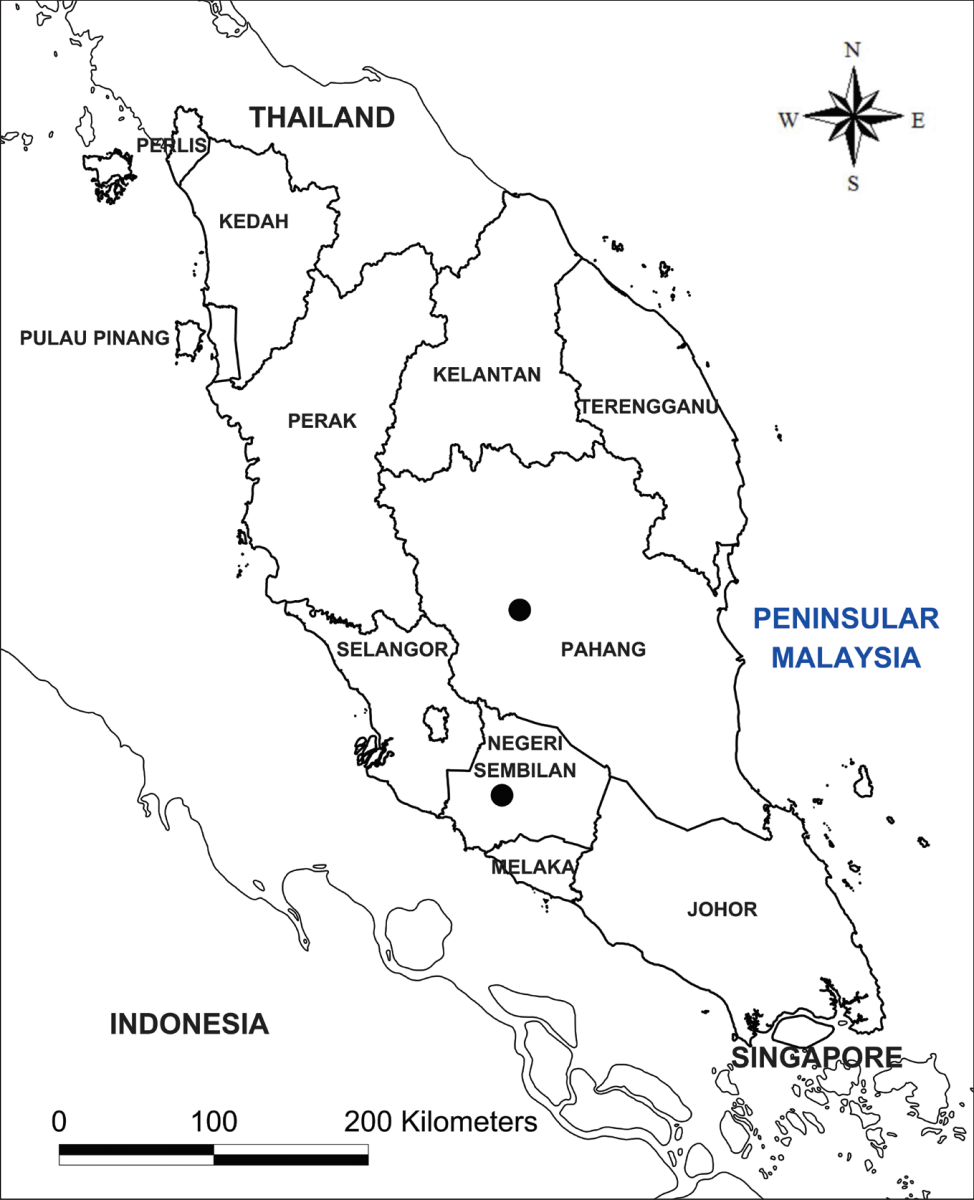
Distribution of *Thismiamalayana* (black circle) in Peninsular Malaysia: the type locality in Ulu Bendul RP, Gunung Angsi FR in the state of Negeri Sembilan and the Tengku Hassanal WR, Temerloh in the state of Pahang.

The specimens of the new species were collected from both localities (Map 1), preserved in 70% ethanol and deposited in the Kepong Herbarium (KEP). Morphological characters were examined, and measurements were made using an Olympus SZ61 stereomicroscope and high-resolution macrophotography of fresh and preserved material. The morphology of the new species was carefully compared with all related species of the genus *Thismia*. Assessment of the conservation status according to the categories and criteria of the IUCN Red List (IUCN Standards and Petitions Committee 2022).

## ﻿Taxonomic account

### ﻿ Thismia malayana

Taxon classificationPlantaeDioscorealesThismiaceae

Siti -Munirah, Hardy-Adrian, Mohamad-Shafiq & Irwan-Syah sp. nov.

103ABA9B-D6C0-5B49-8F1F-7FD4029F2D08

urn:lsid:ipni.org:names:77342735-1

Figs 1 , 2 , 3

#### Diagnosis.

*Thismiamalayana* resembles *T.chrysops* but differs by the brown-whitish colour of the floral tube (vs. very dark sepia brown), glabrous surface of the tepal appendages (vs. finely ciliate), presence of transverse bars at inner surface of floral tube (vs. bars absent), supraconnective apex of each stamen with 5 appendages: one pair of club-shaped inwards-pointing, one pair of acute outwards-pointing, and one central appendage (vs. 7 appendages: one pair of club-shaped, one pair of acute and 3 central appendages).

**Figure 1.**
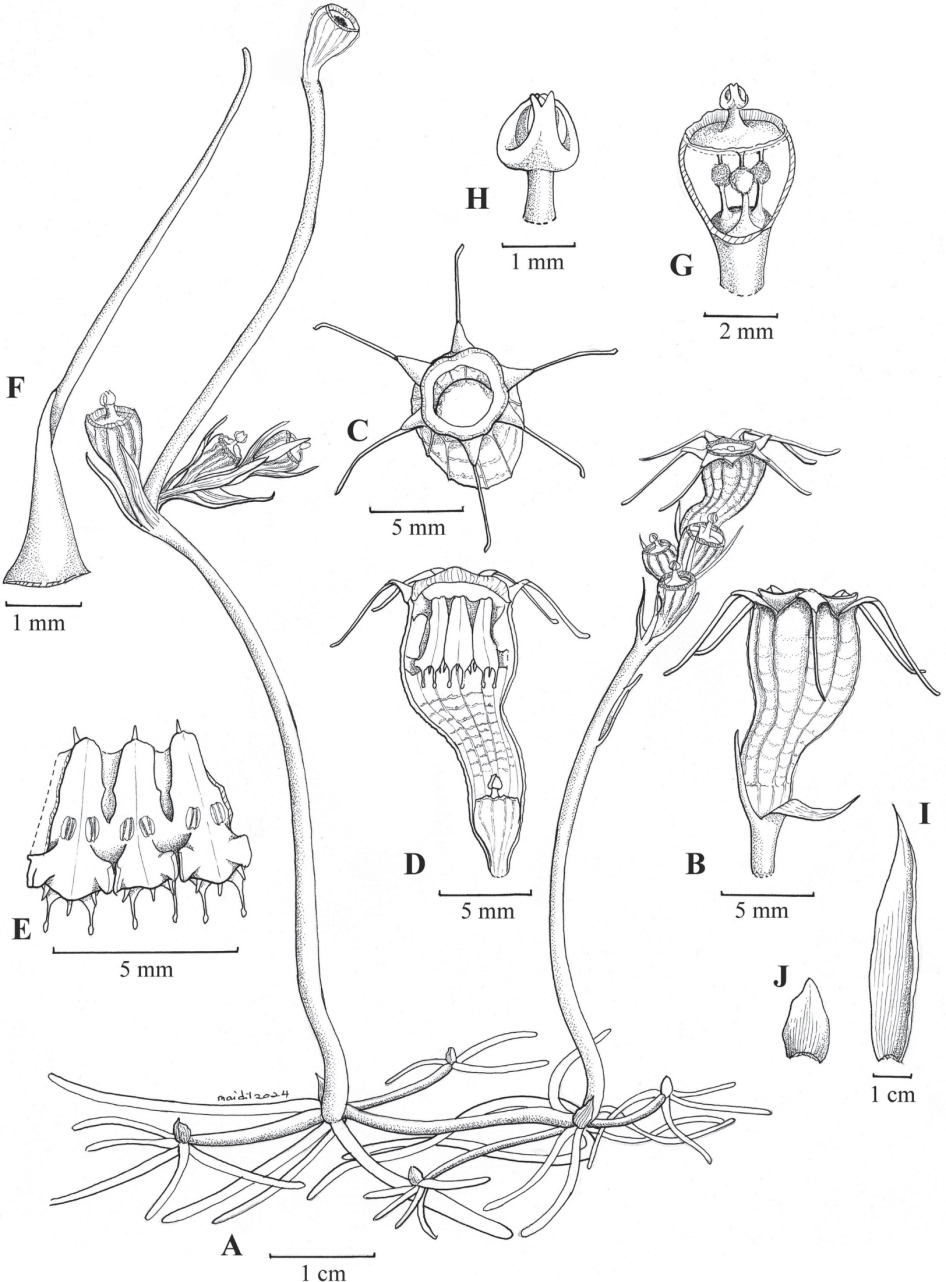
*Thismiamalayana***A** flowering plant **B** flower, side view **C** flower, view from above **D** flower, longitudinal section **E** three stamens, outer view **F** tepal with terminal appendage, adaxial view **G** ovary, longitudinal section **H** style and stigma **I** involucral bract, adaxial view **J** leaf, adaxial view. All drawn by Mohamad Aidil Noordin from spirit material, *Siti-Munirah FRI 101705* (**A–C**); *FRI 101701* (**D–J**).

#### Type.

Malaysia. Peninsular Malaysia: Negeri Sembilan, Kuala Pilah Distr., Gunung Angsi FR, Ulu Bendul RP, a trail to Gunung Angsi., elevation ca. 206–208 m a.s.l., 9 February 2023. *Siti-Munirah* FRI 101705 (holotype KEP!, spirit collection, barcode no. SC12021]).

**Figure 2.**
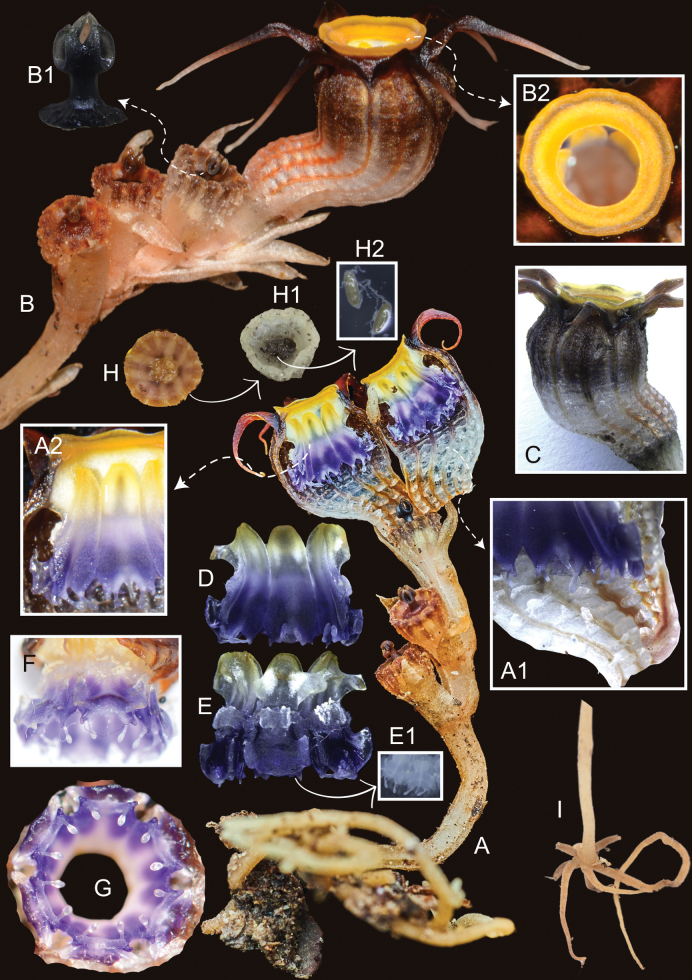
*Thismiamalayana***A** flowering plant **A1** floral tube, inner surface **A2** annulus and stamen filaments, view from inside **B** inflorescence with anthetic flower and several young fruits **B1** style and stigma **B2** annulus, top view **C** flower, side view **D, E** stamens, view from inside and from outside, **E1** stamen supraconnectives: one pair of club-shaped inwards-pointing, one pair of acute outwards-pointing, and one central appendage **F** stamen supraconnectives, apical view **G** stamen tube, view from below **H, H1** fruit after dehiscence, top view, **H2** seeds **I** shoot base with roots. Photos by Siti-Munirah (**A1–I**) and Hardy-Adrian (**A**) from *FRI 101701* (**A**), *FRI 101702* (**F, G, I**), *FRI 101703* (**E1**), *FRI 101705* (**B, B2, H, H1, H2**) & *FRI 101710* (**A1, A2, B1, C, D, E**). Images not to scale (see dimensions in description and Figs 1, 3).

#### Description.

Achlorophyllous herb, up to 10 cm tall, predominantly glabrous. ***Roots*** vermiform, unbranched, ca. 1 mm in diameter, light brown. ***Stem*** erect, up to ca. 6 cm long, 0.2 cm in diameter, pale brownish, bearing 1–4 flowers. ***Leaves*** 2–4, alternate, distant, triangular to narrowly triangular, scale-like, apex acute, margin slightly irregularly serrate (almost entire), 6 mm long, ca. 1 mm wide at base, pale brown. ***Involucral bracts*** 3, spirally/alternately arranged, similar to leaves but slightly larger, triangular to narrowly triangular, scale-like, apex acute, margin entire, 8 mm long, ca. 1.5–2.5 mm wide at base, pale brown or pale orange. ***Pedicel*** ca. 2 mm long at anthesis, elongating to ca. 5 cm long after anthesis, pale brown. ***Flowers*** terminal, asymmetrical, slightly zygomorphic, ca. 13 mm long (including ovary). ***Floral tube*** urceolate-curved, ca. 7 mm long, ca. 5 mm wide at middle, ca. 4 mm wide at base, sigmoidally bent in lower part; ***outer surface*** brown to whitish-sepia (whitish proximally & brown distally), with 12 longitudinal ribs orangish proximally & dark brown distally; ***inner surface*** reticulate, with whitish transverse bars, of similar color as outer surface. ***Tepals*** 6, free, triangular to ovate, apex acute, up to ca. 2.5 mm long, ca. 1.5–2.6 mm wide at base, dark brown, apically bearing a tentacle-like appendage; appendage narrowing towards apex, each equal in length up to ca. 6 mm long, 0.5 mm wide, whitish towards apex, becoming brownish with age. ***Annulus*** moderately raised, ring-shaped, ca. 5.4 mm in diameter, with ring width ca. 1.2 mm, orange, becoming bright yellow with age, aperture ca. 3.2 mm in diameter. ***Stamens*** 6, pendent from annulus; ***filaments*** free, ca. 1 mm long, curved downwards, yellowish to whitish; anther ca. 0.7 mm long; ***connectives and supraconnectives*** narrow at base (ca. 1.5 mm wide) and broad at apex (ca. 2 mm wide), with outer side bluish to violet; inner side bluish, violet, whitish to yellowish, flattened at inner surface, laterally connate to form a tube, ca. 4.5 mm long, supraconnective apex with one pair of club-shaped inwards-pointing, ca. 0.8–1 mm long and one pair of acute outwards-pointing ca. 0.3 mm long appendages, and one central appendage ca. 0.5–0.6 mm long; supraconnective bearing a skirt-like lateral appendage at outer side protruding towards inner side of floral tube, bearing trichomes at both sides; lateral appendage not exceeding the tip of the supraconnective appendages, margin lobed with translucent trichomes; ***interstaminal glands*** inserted on the line of fusion between supraconnectives at the level of attachment of lateral appendages, bluish (concolorous with supraconnectives). ***Ovary*** inferior, unilocular; ***placentas*** 3, free, column-like, arising from the bottom of the ovary; ovules numerous. ***Style*** ca. 0.74 mm long, dark blackish; ***stigma*** ca. 0.85 mm long, papillose, 3-lobed, with lobes ± rectangular and bifurcate at apex, dark blackish. ***Fruit*** dehiscent, cup-shaped, 6 mm in height, 6–8 mm in diameter, pale white to creamy. ***Seeds*** long oval, ca. 0.38–0.41 mm long, ca. 0.15–0.17 mm wide.

#### Additional specimens examined.

Malaysia. Peninsular Malaysia: Negeri Sembilan, Kuala Pilah District, Gunung Angsi FR, Ulu Bendul RP, elev. ca. 206–208 m, February 2023, *Siti-Munirah, FRI 101701* (KEP), *FRI 101702* (KEP), *FRI 101703* (KEP), *FRI 101704* (KEP) *FRI 101709* (KEP), *FRI 101710* (KEP), *FRI 101711* (KEP); Pahang, Temerloh District, Tengku Hassanal WR, elev. ca. 200 m, 31 December 2020, *Mohamad-Shafiq & Irwan-Syah, FRI 91126* (KEP).

**Figure 3.**
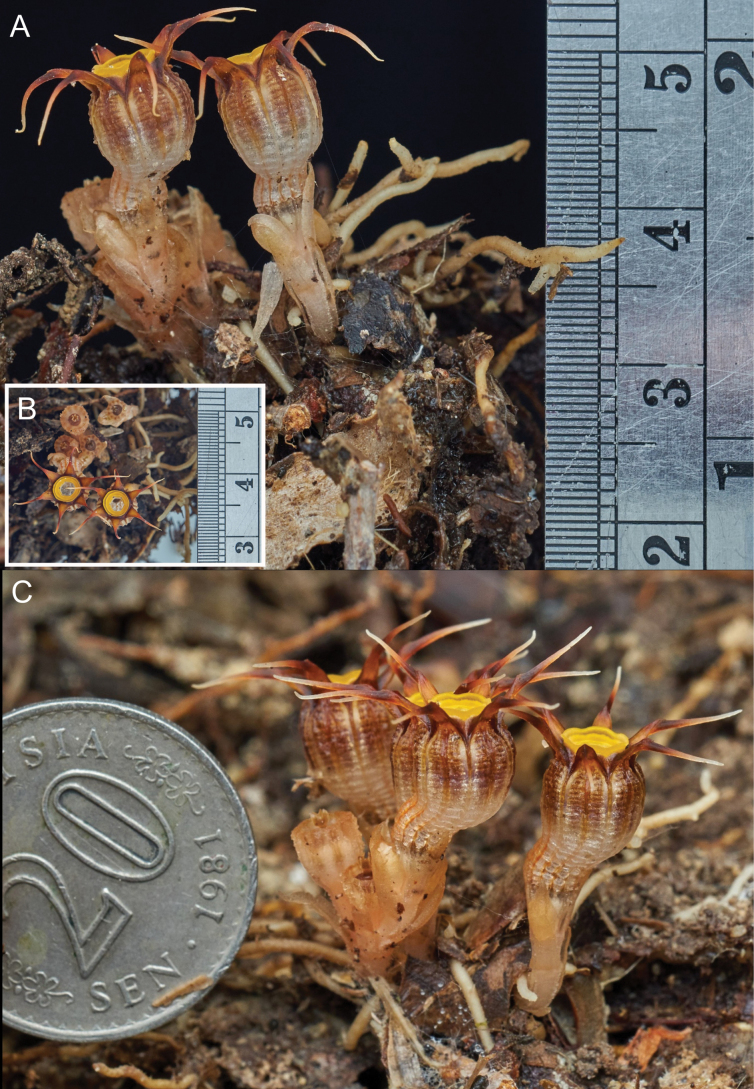
*Thismiamalayana* with scales (the finest grade is 0.5 mm) **A** side view **B** top view **C** the size compared to the 20-sen coin (23.59 mm in diameter). Photos by Hardy-Adrian from uncollected plants.

#### Distribution.

**Figure 4.**
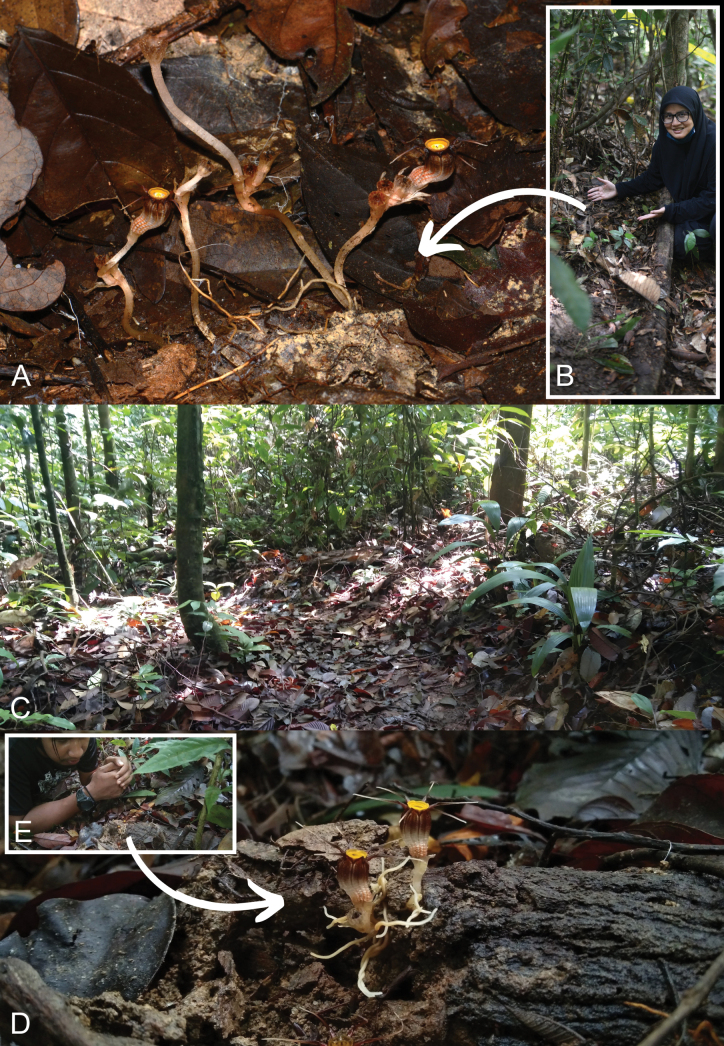
Habitat (in situ) of *Thismiamalayana* in Ulu Bendul RP in Gunung Angsi FR (**A, B**) and the Tengku Hassanal WR (**C–E**) **A***Thismiamalayana* at its habitat, which is located right next to the main trail to Gunung Angsi **B** Siti-Munirah showing the habitat of *T.malayana***C** path to Lata Bujang and Gunung Benom **D** the plants growing on rotten wood **E** Mohamad-Shafiq observed a *Thismiamalayana* in its habitat. Photos by Siti-Munirah (**A, B**) and Mohamad-Shafiq (**C–E**).

Endemic to Peninsular Malaysia. Recorded in two localities: one in Gunung Angsi FR in Negeri Sembilan state and another in Tengku Hassanal WR in Pahang state (Fig. 4).

#### Ecology.

In medium-moist or mesic, shady areas of lowland dipterocarp forests at elevations of 200–450 m a.s.l. Flowering and fruiting usually from December to February but flowering also observed in June. According to the third and fourth author’s observations, *T.malayana* was only sighted and observed a few times a year, especially during the rainy season.

#### Etymology.

The specific epithet *malayana* is derived from Malaya, a name formerly used for Peninsular Malaysia. The epithet refers to the known region of the species distribution.

#### Conservation status.

According to the IUCN standards (IUCN 2022), we propose to classify the preliminary conservation status of *Thismiamalayana* as VU (Vulnerable). This particular species has only been found in two locations. One of the sites is located in a protected area (Tengku Hassanal WR), the other in a water catchment forest under the PRF class protection forest (Gunung Angsi FR), which also includes Ulu Bendul RP. Both habitats are therefore considered stable. However, the population of this species was detected near a hiking trail at both sites, which exposes this species to the risk of trampling due to its small size. During the survey conducted between 2022 and 2023, only less than 10 individuals were found. However, due to the limited time and area of the survey and also mycoheterotrophic nature of the species which remains hidden in the soil for most of its life, it was not possible to determine the estimated number of mature individuals in the population. Therefore, this species is classified as Vulnerable based on criterion D2.

#### Notes.

According to the classifications of Jonker (1938) and Kumar et al. (2017), *T.malayana* is placed in subgenus ThismiasectionThismiasubsectionOdoardoa Schltr. (Schlechter 1921), as it has six free, equal tepals and vermiform roots. Among the ThismiasubsectionOdoardoa, the following species share the curved or bent floral tube characteristic of *T.malayana*: *T.chrysops* from Johor (Gunung Ledang, Peninsular Malaysia) (Ridley 1895), *T.cornuta* from Sarawak (Malaysia) (Hroneš et al. 2018), *T.inconspicua* from Brunei (Sochor et al. 2017) and *T.kinabaluensis* from Sabah (Malaysia) (Nishioka et al. 2018). A detailed morphological comparison of *T.malayana* and these morphologically most similar four species is presented in Table 1.

**Table 1.** Morphological comparison of *T.malayana* with *T.chrysops* (Ridley 1895), *T.cornuta* (Hroneš et al. 2018), *T.kinabaluensis* (Nishioka et al. 2018) and *T.inconspicua* (Sochor et al. 2017).

Characteristics	*T.malayana*	*T.chrysops*	*T.cornuta*	*T.inconspicua*	*T.kinabaluensis*
**Floral tube**					
**Outer**					
colour	brown to whitish-sepia	very dark sepia brown	translucent-white	(light) brownish	brown or beige
colour of longitudinal ribs					
upper part	dark brown in	rose pink	pale pinkish	sepia-brown	light brown or beige streaks
lower part	orangish	chocolate-brown	pale pinkish	both ribs and background gradually darkening toward the apex	of the same colour as the floral tube
**Inner**					
transverse bars	present	absent	absent	absent	present
**Annulus**					
colour	bright yellow	bright yellow	pinkish	sepia-brown on the outer margin, brownish-orange to light orange on the inner margin and grayish in between	with blue circle on the outer margin, orange to yellow on the inner margin
**Tepal**					
colour	dark brown	bright sienna, brown/ chocolate brown	white	sepia-brown	pale blue
**Appendages**					
colour	brown to whitish	bright sienna brown	white	sepia-brown	pale blue
surface	glabrous	finely ciliate	glabrous	glabrous	glabrous
**Stamens**					
colour	bluish, violet, whitish, yellowish	unknown	translucent-white	translucent-white	translucent-white
appendages of supraconnectives	5 (2 club-shaped pointing centripetally, 2 acute pointing centrifugally and 1 central appendage)	several, (2 club-shaped and few shorter ones)	2 club-shaped	4 (2 club and 2 tooth-shaped)	3 (1 filiform between 2 club-shaped)

## Supplementary Material

XML Treatment for Thismia malayana

## ﻿Additional information

### Author ORCIDs

Mat Yunoh Siti-Munirah https://orcid.org/0000-0002-5062-9988

### Data availability

All of the data that support the findings of this study are available in the main text.

## References

[B1] Davis SD Heywood VH Hamilton AC [Eds] (1995) Centres of plant diversity: A guide and strategy for their conservation, II. Asia, Australasia and the Pacific. Worldwide Fund for Nature (WWF) and IUCN (The World Conservation Union), IUCN Publications, University of Cambridge, [xiv +]578 pp.

[B2] Feller B Dančák M Hroneš M Sochor M Suetsugu K Imhof S (2022) Mycorrhizal structures in mycoheterotrophic *Thismia* spp. (Thismiaceae): Functional and evolutionary interpretations. Mycorrhiza 32(3–4): 269–280. 10.1007/s00572-022-01076-3 35419710 PMC9184416

[B3] Hroneš M Rejžek M Sochor M Svátek M Kvasnica J Egertová Z Pereira JT Nilus R Dančák M (2018) Two new species of Thismiasubsect.Odoardoa (Thismiaceae) from Borneo. Plant Ecology and Evolution 151(1): 110–118. 10.5091/plecevo.2018.1387

[B4] IUCN Standards and Petitions Committee (2022) Guidelines for Using the IUCN Red List Categories and Criteria. Version 15.1. Prepared by the Standards and Petitions Committee. https://www.iucnredlist.org/documents/RedListGuidelines.pdf

[B5] Jonker FP (1938) A monograph of the Burmanniaceae. Mededeelingen van het Botanisch Museum en Herbarium van de Rijks Universiteit te Utrecht (Utrecht) 51: 1–279.

[B6] Kumar P Gale SW Li J Bouamanivong S Fischer GA (2017) *Thismianigricoronata*, a new species of Burmanniaceae (Thismieae, Dioscoreales) from Vang Vieng, Vientiane Province, Laos and a key to subgeneric classification. Phytotaxa 319(3): 225–240. 10.11646/phytotaxa.319.3.2

[B7] Leake JR (1994) The biology of myco-heterotrophic (saprophytic) plants. The New Phytologist 127(2): 171–216. 10.1111/j.1469-8137.1994.tb04272.x 33874520

[B8] Merckx VSFT (2013) Mycoheterotrophy: The biology of plants living on fungi. Springer, New York, NY. 10.1007/978-1-4614-5209-6

[B9] Nishioka T Suetsugu K Repin R Kitayama K (2018) *Thismiakinabaluensis* (Thismiaceae), a new species from Mt. Kinabalu, Sabah, Borneo. Phytotaxa 360(2): 174–178. 10.11646/phytotaxa.360.2.10

[B10] POWO (2024) Plants of the World Online. Facilitated by the Royal Botanic Gardens, Kew. http://www.plantsoftheworldonline.org/ [Retrieved 30 March 2024]

[B11] Ridley H (1895) Two new species of *Thismia.* Annals of Botany 9(2): 323–326. 10.1093/oxfordjournals.aob.a090741

[B12] Schlechter FRR (1921) Die Thismieae. Notizblatt des Botanischen Gartens und Museums zu Berlin-Dahlem 8: 31–45. 10.2307/3994560

[B13] Sochor M Sukri RS Metali F Dančák M (2017) *Thismiainconspicua* (Thismiaceae), a new mycoheterotrophic species from Borneo. Phytotaxa 295(3): 263–270. 10.11646/phytotaxa.295.3.7

[B14] Thorogood CJ Siti-Munirah MY (2021) Fairy lanterns in focus. Plants, People, Planet 3(6): 680–684. 10.1002/ppp3.10217

[B15] Watkinson SC (2016) Mutualistic Symbiosis Between Fungi and Autotrophs. The Fungi: 205–243. 10.1016/B978-0-12-382034-1.00007-4

